# Enzymatic Bioremediation of Organophosphate Compounds—Progress and Remaining Challenges

**DOI:** 10.3389/fbioe.2019.00289

**Published:** 2019-11-08

**Authors:** Meghna Thakur, Igor L. Medintz, Scott A. Walper

**Affiliations:** ^1^College of Science, George Mason University, Fairfax, VA, United States; ^2^Center for Bio/Molecular Sciences, U.S. Naval Research Laboratory, Washington, DC, United States

**Keywords:** organophosphate, enzyme, chemical warfare agent, bioremediation, catalysis, outer membrane vesicle, decontamination, phosphotriesterase

## Abstract

Organophosphate compounds are ubiquitously employed as agricultural pesticides and maintained as chemical warfare agents by several nations. These compounds are highly toxic, show environmental persistence and accumulation, and contribute to numerous cases of poisoning and death each year. While their use as weapons of mass destruction is rare, these never fully disappear into obscurity as they continue to be tools of fear and control by governments and terrorist organizations. Beyond weaponization, their wide-scale dissemination as agricultural products has led to environmental accumulation and intoxication of soil and water across the globe. Therefore, there is a dire need for rapid and safe agents for environmental bioremediation, personal decontamination, and as therapeutic detoxicants. Organophosphate hydrolyzing enzymes are emerging as appealing targets to satisfy decontamination needs owing to their ability to hydrolyze both pesticides and nerve agents using biologically-derived materials safe for both the environment and the individual. As the release of genetically modified organisms is not widely accepted practice, researchers are exploring alternative strategies of organophosphate bioremediation that focus on cell-free enzyme systems. In this review, we first discuss several of the more prevalent organophosphorus hydrolyzing enzymes along with research and engineering efforts that have led to an enhancement in their activity, substrate tolerance, and stability. In the later half we focus on advances achieved through research focusing on enhancing the catalytic activity and stability of phosphotriesterase, a model organophosphate hydrolase, using various approaches such as nanoparticle display, DNA scaffolding, and outer membrane vesicle encapsulation.

## Introduction

Biological diversity spread across the ecological niches of the world has allowed for the evolution of cellular systems that enable the survival of microbes, plants, and animal in these highly variable environments. This has been recognized at the phenotypic level since the time of Charles Darwin's voyage on the Beagle. Today we characterize Darwinian evolutions at the genetics level recognizing that such adaptability occurs through alterations in cellular physiology or mechanisms, many of which are the product of adapted or novel enzymatic pathways and the products they produce. Examples such as the bacteria found in arsenic contaminated aquifers or the fungi capable of growing within nuclear reactors highlight the ability of Nature to adapt to harsh environments and exploit available resources to survive (Dadachova and Casadevall, 2008; Gnanaprakasam et al., 2017; Gu et al., 2017). In addition to basic survival mechanisms, the endless quest for the raw materials necessary to sustain life has also allowed for organisms to develop metabolic pathways allowing them to scavenge, convert, and utilize an incredible range of both natural and man-made chemicals to satisfy these needs. The cellular tools of these conversions, enzymes, are capable of chemical conversions and transformations that occur incredibly fast, often at the rate of diffusion, and with a specificity and selectivity that cannot easily be rivaled using chemical catalysis. For this reason, enzymes are rapidly developing as invaluable tools in medicine as therapeutics, as agents of environmental decontamination, and as reagents that enable a diverse spectrum of commercial applications.

Historically, mankind exploited cellular processes such as fermentation for the preparation of food and drink. Today, advances and understanding in cellular and metabolic processes has allowed for an explosion in the use of purified enzymes within the food industry (Raveendran et al., 2018). Similarly, the use of enzyme-based therapeutics continues to rise as these biomolecules typically show reduced toxicity and immunogenicity compared to other chemically synthesized alternatives (Dean et al., 2017; Kumar and Abdulhameed, 2017; Yari et al., 2017). Enzymes for commercial and industrial applications is also on the rise as these biomolecules offer a sustainable path with reduced toxic waste products (Chapman et al., 2018). One area of particular interest in research and development is in the area of bioremediation and detoxification of chemicals.

**Figure 1:**
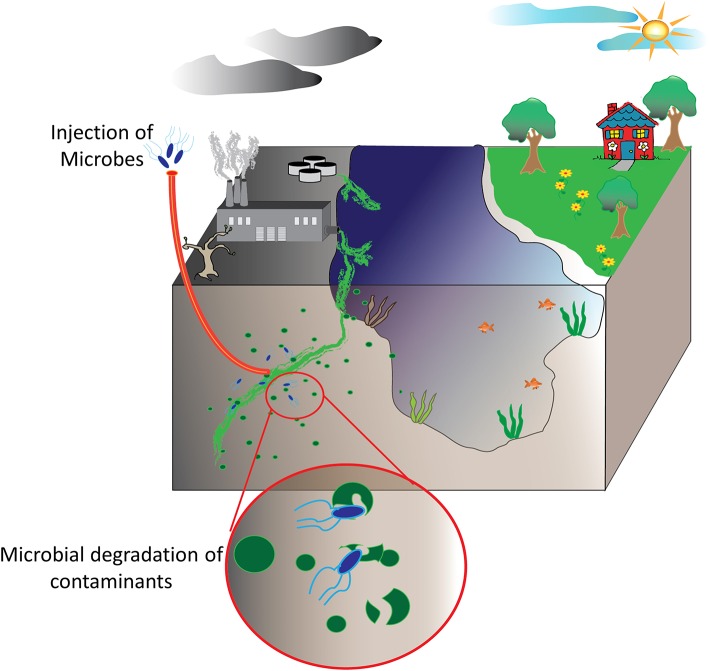
Bioremediation of environmental pollutants. Environmental decontamination of industrial waste products and other contaminants, ideally, is realized by microbes and plant species able to convert these toxic compounds into other biomolecules that these utilize for their own metabolic functions.

Bioremediation at its most basic definition is the use of natural biological systems such as plants and microbes to degrade or consume environmental pollutants. The potential of bioremediation was recognized decades ago starting with efforts to treat wastewater using environmental isolates. The first documented success with environmental microbes was detailed in a 1975 report by Raymond et al. that describes the degradation of petroleum-derived hydrocarbons by microbial populations (Raymond et al., 1975; Dvorak et al., 2017). Expanding on these studies, researchers have sought to harness this phenomenon for other purposes, particularly environmental decontamination. Contaminants such as industrial waste products including polycyclic aromatic compounds, organic dyes, heavy metals, and polyhalogenated compounds are common contaminants in aquifers and soil as are petroleum-based hydrocarbons and pharmaceutical compounds. Fortunately, each of these have been targets of various bioremediation strategies (Singh and Walker, 2006; Yi and Crowley, 2007; Peng et al., 2008; Ben Mansour et al., 2012; Montgomery et al., 2013; Rodgers-Vieira et al., 2015; Dzionek et al., 2016; Brar et al., 2017; Dvorak et al., 2017; Ojuederie and Babalola, 2017; Wu et al., 2017; Liu et al., 2018). The adaptability and versatility of plants and microbes has enabled bioremediation as a “biologically-friendly” strategy to eliminate many of the harmful contaminants released by the everyday machinations of human society, see schematic overview in Figure 1. At present, bioremediation of environmental reservoirs is accomplished using primarily natural, non-engineered bacteria that have been isolated from contaminated sources and exhibit an ability to consume or convert the target contaminant as highlighted in several examples (Cologgi et al., 2011; Silar et al., 2011; Dubinsky et al., 2013; Prakash et al., 2013; Gustavsson et al., 2016). With the rise of synthetic biology and microbial engineering, future strategies may likely include engineered microbes containing novel metabolic pathways or tailored for enhanced fitness in austere environments. Despite the advantages these strategies may afford, regulations in the U.S. and across Europe limit or prohibit the use and release of genetically modified organisms into the environment. For this reason, many researchers are investigating the potential direct use of recombinant enzymes for the purpose of environmental decontamination. Often referred to as cell-free synthetic biology, these strategies offer a fine level of control where researchers can tailor reaction conditions such as enzyme concentrations, the availability of co-factors, ionic strength, and others. Of significant importance, unlike the engineered microbes from which they are derived, cell-free catalytic systems are non-replicative and are therefore less likely to be impacted by regulatory policies.

This review will focus on enzymatic systems for the bioremediation of a ubiquitous but highly dangerous group of man-made compounds collectively referred to as organophosphates (OPs). First described in the late nineteenth century, these compounds existed in relative obscurity until their potential as anti-biological agents were recognized during World War II. Since then they have rapidly evolved from chemical warfare agents (CWAs) to commercial pesticides used all over the world. As both agents of war and agricultural tools, these chemical compounds have left a lasting effect on the environment as they are contaminants of both soil and water capable of inducing illness and death to those that inadvertently encounter them. Fortunately, microbial species have rapidly evolved enzymatic processes to degrade these contaminants and researchers have developed strategies to utilize these biological catalysts for bioremediation of OP compounds. In this review we will detail many of the enzymes currently employed and studied for OP remediation describing the properties of each and efforts to develop them into products useful for environmental clean-up. Unfortunately, recombinant enzymes often suffer from issues that limit their wide-spread use as a viable material. However, as the tools and techniques of biological engineering continue to expand, the utility of enzymes as therapeutics and implements of decontamination of organophosphate compounds has been reinvigorated. This review will describe how new methods of biological engineering such as synthetic biology and cell-free synthetic biology are breathing new life into enzyme-based catalytic systems for organophosphate decontamination and neutralization. Here we pay particular attention to efforts to enhance the catalytic activity, substrate promiscuity, and stability of the more commonly used OP hydrolyzing enzymes through enzyme engineering, assembly of scaffolded cell-free systems, and through the development of microbe-based systems of bioremediation that revolve around the re-engineering of bacterial genetic circuits and cellular systems. Each of these areas highlights how the engineering of biology at the protein, system, and organism level can be utilized to address issues such as environmental contamination that affect populations around the globe.

## Organophosphates

**Figure 2:**
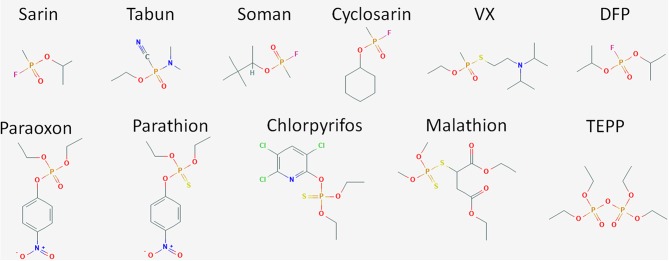
Structure of OP compounds. The diversity of OP compounds is highlighted here in the chemical structures of some of the better known CWA nerve agents (sarin, tabun, soman, cyclosarin, VX, and DFP) and pesticides (paraoxon, parathion, chlorpyrifos, trichlorfon, dichlorvos, malathion, TEPP).

Organophosphate compounds are typically phosphate esters formed from a reaction between alcohols and phosphoric acid resulting in a compound with the general structure of O = P(OR)_3_. OP compounds are found in both naturally occurring biomolecules and industrial products such as pesticides including insecticides and herbicides. The first of these compounds was chemically synthesized in the late nineteenth century by Phillipe de Clemont and Wladimir Moschnin, but it was not until much later, in 1932, that Willy Lange and his student Gerde von Krueger described the cholinergic effects of these compounds (Costa, 2018; Franjesevic et al., 2019). Over the next 20 years, British and German scientists produced a suite of OP compounds that could be used as highly potent nerve agents. Later, American scientists and others adapted these chemical syntheses to manufacture OP insecticides. Chemical structures for some of the more prevalent and toxic OP compounds are shown in Figure 2. Toxicity of these compounds is highly variable and dependent on the chemical composition, the route of entry or exposure, and whether exposure is acute or chronic (Munro et al., 1999; Costa, 2018). While not as lethal as their CWA counterparts, OP pesticides can also be quite dangerous capable of causing illness or death. For both pesticides and CWAs, the ease and low cost of production paired with the high degree of efficacy has ensured that OP compounds continue to find use in both beneficial and nefarious applications.

**Figure 3:**
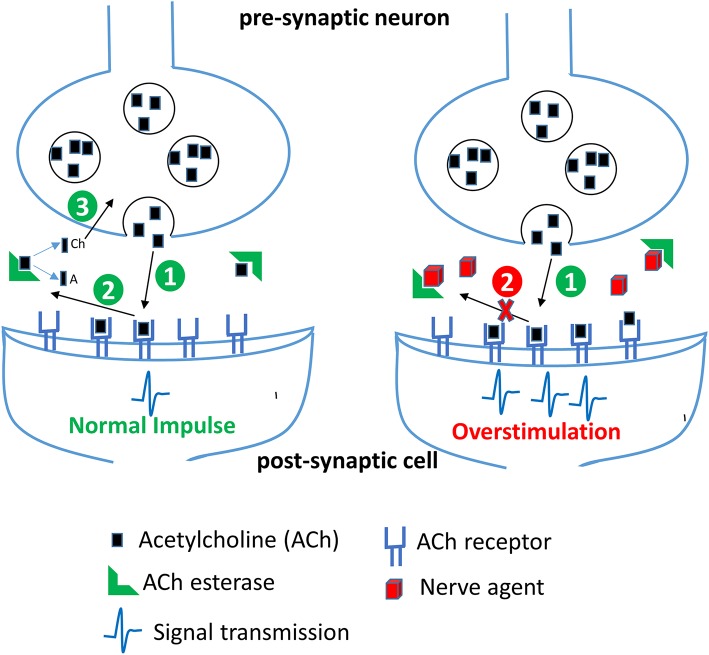
Effect of nerve agents on central nervous system. Acetylcholine (ACh) released from the synaptic vesicles in the presynaptic neuron binds to its receptor in the postsynaptic membrane resulting in generation of a nerve impulse (step 1). In the second step, ACh dissociates from its receptor in the synaptic cleft and is rapidly broken down by the enzyme acetylcholinesterase (AChE) into acetate (A) and choline (Ch). Choline is transported back into the axon terminal and is used to make more ACh. In the presence of nerve agents, AChE is irreversibly inhibited, thereby resulting in an accumulation of ACh and a persistent stimulation of ACh receptors leading to continuous neurotransmission and an acute cholinergic syndrome.

OP compounds were rapidly adopted as CWAs first by the Nazis and subsequently by the British during World War II to replace the chlorine-based weapons of the previous World War. The German efforts during this time period produced the G-series of agents including sarin, cyclosarin, and soman. In parallel, the British developed a series of reagents including diisopropylfluorophosphate (DFP), VX, and VR. While diverse in chemical composition and structure, CWAs and OP pesticides function through the disruption of the enzyme acetylcholinesterase, a critical component of signal transduction in the neuromuscular junctions of both insects and animals, see schematic in Figure 3. Accumulation of acetycholine, a neurotransmitter, in the synaptic cleft leads to overstimulation of acetylcholine receptors thereby causing a cholinergic crisis characterized by anxiety, headache, convulsion, tremor and death (Jokanovic, 2018; Naughton and Terry, 2018; Sikary, 2019). Though developed during the Second World War these compounds did not see use until many years later in other conflict areas. Sarin saw widespread use by the Iraqi army during the Iran-Iraq conflicts of the 1980s resulting in the deaths of thousands despite international treaties condemning the use of CWAs. Later, in 1995, the Japanese doomsday cult Aum Shinrikyo deployed sarin gas on a Tokyo subway train killing 13 and injuring or affecting nearly 1,000 others. The same group also experimented with the production and use of the even more toxic organophosphate CWA, VX (Zurer, 1998). These compounds continue to find nefarious use today. In 2013 and again in 2017, the Syrian government bombarded cities within their own borders with sarin gas to quell a growing rebellion. OP nerve agents have also been used for assassinations as shown in the 2017 slaying of Kim Jong-nam a half-brother of the current North Korean leader and the attempt on the life of a former Russian spy, Sergei Skripal in 2018.

While governments and terrorist organizations have employed OPs as weapons of mass destruction and terror, these compounds have also seen widespread use in agriculture in both developed and developing countries. During the twenty-first century, OPs were the most widely used pesticides in the United States with as many as 36 compounds approved for domestic or agricultural use as summarized in Supplemental Table 1 (Roberts and Reigart, 2013). It is estimated that millions of tons of these compounds are produced and dispersed each year in the U.S. alone (Atwood and Paisley-Jones, 2017). The ubiquitous utilization of these compounds leads to a high rate of human exposure and OP poisoning. It is estimated that around 3,000,000 people are exposed to OP compounds per year accounting for more than 300,000 fatalities (Bird et al., 2019). Despite the well-documented acute and chronic toxic effects, OPs are seen as vital components of commercial agriculture and pest control (Knutson and Smith, 1999; Jokanovic, 2018; Lushchak et al., 2018; Mostafalou and Abdollahi, 2018). However, in developed countries there is growing concern for the long-term use of these compounds and their environmental accumulation and toxicity. As an example, chlorpyrifos, which was first registered with the U.S. Environmental Protection Agency (EPA) in 1965, has seen a gradual shift in policy regarding its use beginning in 1996 with phase out of home use and in some agricultural products such as tomatoes. Since then the EPA has continued to examine this OP and its environmental and health effects modifying policy for its use. Mostly recently these studies and others have resulted a 2018 decision by the U.S. Ninth Circuit Court of Appeals ordering an EPA-approved ban of the compound (https://www.epa.gov/ingredients-used-pesticide-products/chlorpyrifos). Continuous studies of OP compounds and events such as the death of 25 school children in India from meals contaminated with monocrotophos, an agricultural pesticide, has led to increased public attention which in turn has fueled scientific research in methods of ensuring public safety through the development of new safer pesticides and methods of remediating OPs from the environment (Banerji et al., 2013; Than, 2013).

## Decontamination of Organophosphates

The reactive nature of organophosphate compounds has allowed for the development of numerous strategies to remove and/or neutralize these materials from the environment. Typically, decontamination of organophosphates, both pesticides and CWAs, is accomplished through (1) physical removal or dilution of the material through the use of absorbents or flushing contaminated areas, (2) chemical decontamination, or (3) bioremediation using microbial species or purified enzymes (Jacquet et al., 2016). Ideally, several of these methods would be used in conjunction to detoxify target environments, equipment, and infrastructure (see Table 1).

**Table 1:** Common methods of decontamination and their application.

**Method**	**Applications**	**Advantages**	**Disadvantages**
*Physical*			
Incineration	Elimination of stockpiles	Ease of use and efficiency	May require transport of material (expense), potential for volatile release
Landfill, Flushing	Elimination of stockpiles	Ease of use Inexpensive	Soil and aquifer contamination
*Chemical*	Elimination of stockpiles, equipment and material decon (some), personnel decon (limited)	Ease of use and efficiency Rapid decon	Expensive, potentially corrosive agents damaging equipment, secondary contamination due to catalyst or agent
*Bioremediation*	Elimination of stockpiles, equipment and material decon, personnel decon	Bio-friendly (non-toxic) Highly efficient with limited secondary contamination	Regulatory concerns, expensive of enzyme production, stability and shelf-life (enzymes), low readiness level

### Physical Decontamination

Physical decontamination of contaminated areas involves the use of incineration, sorbent materials, or simply burying or washing contaminated surfaces to minimize the risk of exposure. Unfortunately, this does not typically neutralize the material which can lead to secondary contamination particularly of soils and aquifers. Materials for this type of decontamination can span the gamut from nanoparticles to bulk use of minerals in material platforms. For example, to address aquifer contamination with OP pesticides Liu et al. developed graphene-coated silica nanoparticles capable of rapidly capturing nine different pesticides from spiked water samples (Liu et al., 2013). Other reactive materials such as cerium dioxide have shown success against both pesticides and CWAs. In laboratory trials, the research team of Lubos Vrtoch showed that cerium dioxide sorbent materials could be used to rapidly degrade both pesticides and CWAs such as VX (Janos et al., 2014). In these studies the group showed that methyl parathion, soman, and VX could be degraded in as little as 30 min after exposure using their cerium dioxide sorbent materials. Unfortunately, while cerium dioxide does have potential as a decontamination strategy studies have shown that exposure to these materials can cause immunostimulation in laboratory models and can lead to toxicity in some human cell lines (Gehlhaus et al., 2009; Mittal and Pandey, 2014).

### Chemical Decontamination

Chemical reagents facilitating the hydrolysis of pesticides and CWAs as a route of decontamination are by far the most prevalent and include hydrolysis, oxidation, and reduction mechanisms. At present, materials relying on chemical hydrolysis are most commonly used for large-scale remediation of contaminated areas (Jacquet et al., 2016). Common reagents such as sodium hydroxide and hypochlorite will react with many OP compounds though both of these compounds are corrosive and not suitable for personal use or with sensitive materials (Dowling and Lemley, 1992; Tuorinsky et al., 2009; Kitamura et al., 2014). Commercial products such as Decontamination Solution 2 (DS2), BX-24, and Decontamination Formulation DF-200 are employed by U.S. and NATO forces and are highly effective against most organophosphate compounds. As with sodium hydroxide and hypochlorite both DS2 and DF-200 are composed of chemicals that limit their use for personnel or sensitive equipment due to their corrosive nature. Given warfighter and support personnel needs, personnel decontamination solutions have also been developed including the M291 Skin Decontamination Kit (SDK) and the Reactive Skin Decontamination Lotion (RSDL). These products have variable effectiveness toward organophosphate agents and neither are suitable for wound or eye decontamination. These materials and other emerging chemical decontamination strategies are thoroughly reviewed in Jacquet et al. (2016).

Decontamination agents such as DS2 and bleach are corrosive in nature and generate hazardous waste and are therefore not considered safe. This leads to a dire need for non-toxic, non-corrosive, and environmentally compatible decontamination strategies. The use of microbes and purified enzymes for decontamination has been an area that is being extensively researched and has met with success in terms of degradation of OP compounds in commercial applications.

### Bioremediation

As will be discussed in subsequent sections, several enzymes have been identified in nature capable of degrading OP pesticides and CWAs (Dawson et al., 2008; Yair et al., 2008; Pizzul et al., 2009; Diao et al., 2013; Iyer et al., 2013; Geed et al., 2016; Brar et al., 2017). Several of these enzymes have been localized to the surface of microbes for evolution of substrate promiscuity and direct bioremediation of OP compounds (Richins et al., 1997; Cho et al., 2002; Zhang et al., 2004; Makkar, 2013; Alves et al., 2015, 2016, 2018; Bigley et al., 2015; Bigely et al., 2019). Cell-mediated bioremediation and biocatalysis is well-established and documented within the literature, however, application can be limited as the release of genetically-modified organisms is not currently considered a viable approach to bioremediation. Additionally, the unique role that OP compounds play as both commercial products and weapons of mass destruction necessitates remediation strategies that can also transition to therapeutic agents and personnel decontamination products.

Over the past several years, numerous successes have been realized with both the isolation of new enzymes capable of degrading OP compounds and the evolution of older enzymes to improve their utility in point-of-concern decontamination. In the subsequent sections we describe those enzymes most commonly referenced in the literature and highlight some of the new players in the field. For each of these enzymes, we also briefly discuss how scientists are attempting to further enable the use of these biological catalyst through protein engineering to increase substrate promiscuity and activity as well as other strategies to improve catalytic activity and stability.

## Enzymes for Decontamination

As previously mentioned, OP-intoxication occurs through the irreversible binding of OP compounds to acetylcholinesterase, an enzyme found within the neuromuscular junction, inactivating it. While acetylcholinesterase is incapable of degrading these compounds, enzyme catalysts capable of degrading OP compounds have been identified in microbial species, in squid, and even in mammals. As will be detailed below, enzymatic degradation of OP compounds occurs through nucleophilic attack of the phosphorus center of the compound mediated by a pair of divalent metal ions, a water molecule, and reactive amino acids contained within the active site of the enzyme (Aubert et al., 2004; Wymore et al., 2014). These enzymes have varying specificities for OP compounds but all follow similar mechanisms of action.

**Figure 4:**
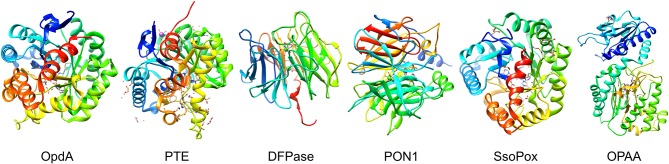
Organophosphate hydrolyases. Representative protein structures from the Protein Data Bank (PDB) database. From left to right: Crystal structure phosphotriesterase OpdA from *Agrobacterium radiobacter* (PDB: 2D2J); crystal structure of zinc-containing phosphotriesterase (PTE) from *Pseudomonas diminuta* (PDB: 1HZY, Chain A); crystal structure of diisopropylfluorophosphatase (DFPase) from *Loligo vulgaris* (PDB: 1E1A); crystal structure of serum paraoxonase PON1 from *Oryctolagus cuniculus* (PDB: 1V04); crystal structure of phosphotriesterase-like lactonase SsoPox from *Sulfolobus solfataricus* (PDB: 2VC5, Chain A); crystal structure of organophosphate acid anhydrolase (OPAA) from *Altermonas sp*. (PDB: 3L7G, Chain A).

An ideal decontaminant candidate must be able to rapidly detoxify the warfare agents at a molecular level and be able to decontaminate any surface such as paint, concrete, rubber seals, asphalt, metal, plastics, clothing, and skin. Additionally, these reagents should be environmentally friendly without lasting impact on soil, vegetation, and animal life or underground water sources. Use of OP degrading enzymes for environmental decontamination present significant advantages as they can rapidly hydrolyze the nerve agents, and are environmental friendly, non-flammable, non-corrosive and can be disposed of safely and easily. Several enzymes have evolved in nature with the capability of degrading the OP compounds, see Figure 4 for names and representative structure and Table 2 for a summary details on each. Extensive research is being carried out to improve their efficacy and stability, thus enhancing their appeal as decontamination agents. Some of these advanced enzymes are being investigated for *in vivo* efficacy, and one of the enzyme based decontamination solutions is already in commercial use in Australia. We now briefly summarize some of the more prominent enzymes studied for OP remediation.

**Table 2:** Summary of enzymes discussed.

**Enzyme**	**Originating species**	**Structure**	**Metal ion**	**Substrates**	**kcat/Km M**^****−1****^**s**^****−1****^		**Notes**	**References**
OpdA	*Agrobacterium radiobacter*	TIM barrel	Binuclear Fe^2+^-Zn^2+^	OPs, G-type nerve agents	Methyl-paraoxon	1.1 X 10^4^	High affinity for substrates with shorter side chains	Horne et al., 2002; Yang et al., 2003
PTE	*Brevundimonas diminuta*	TIM barrel	Zn	OPs, sarin, cyclosarin and VX	DEVX	1.2 X 10^3^	Metal-containing amidohydrolase superfamily	Dumas et al., 1989a; Bigely et al., 2019
					DMVX	1.9 X 10		
					malathion	3.0 X 10		
DFPase	*Loligo vulgaris*	Six-bladed β propellar	Ca^2+^	DFP, G-type nerve agents	DFP	5.6 X 10^4^	Highly specific for P-F bond hydrolysis, high pH and temperature stability	Melzer et al., 2009, 2012; Zhang et al., 2018
					GB	4.2 X 10^4^		
					GF	7.2 X 10^5^		
PON1	Human liver	Six-bladed propeller	Ca^2+^	OPs, G- and V-type nerve agents	Paraoxon	0.6 X 10^4^	High density lipoprotein-associated esterase/lactonase	Aharoni et al., 2004; Purg et al., 2017
Ssopox	S*ulfolobus solfataricus*	TIM barrel	Co^2+^, Fe^3+^	OPs	Paraoxon	4 X 10^3^	Denaturation temperature 106°C	Merone et al., 2005; Elias et al., 2008
OPAA	*Alteromonas sp*.	Pita bread architecture	Mn^2+^	G-agents- soman and cyclosarin	Paraoxon	9.36 X 10^2^	Prolidases, can cleave P-F, P-O, P-CN and P-S bonds	Vyas et al., 2010; Xiao et al., 2017

*OPs, Organophosphates; DEVX, Diethyl-VX; DMVX, Dimethyl-VX; DFP, Diisopropyl fluorophosphate; GB, sarin; GF, cyclosarin*.

### Diisopropylfluorophosphatase (DFPase)

Diisopropylfluorophosphatase (DFPase), isolated from the European squid *Loligo vulgaris*, catalyzes the hydrolysis of OP compounds such as diisopropylfluorophosphate (DFP) and a number of G-type nerve agents, including sarin (GB), soman (GD), cyclosarin (GF), and tabun (GA) (Hartleib and Ruterjans, 2001; Soares et al., 2018). Several structural studies on DFPase have revealed the presence of a six-bladed ß-propeller structure with two calcium ions, one required for catalysis and the other for providing structural integrity (Scharff et al., 2001; Blum and Chen, 2010; Blum et al., 2010). Several theoretical and computational studies in agreement with the experimental results have highlighted the existence of at least two different pathways for the degradation of different OP compounds which could eventually lead to improved strategies to engineer DFPase for more efficient degradation of OP compounds (Soares et al., 2018; Zhang et al., 2018). In addition, mutational studies have also been carried out and have led to an understanding of the mechanism of action of the enzyme on substrates (Blum et al., 2006). Remarkably, in a preliminary *in vivo* study, Melzer *et al*. found that addition of pegylated DFPase minimizes lethality in rats challenged with a subcutaneous 3xLD_50_ dose of soman, thereby highlighting its potential for *in vivo* use (Melzer et al., 2012).

### Paraoxonase (PON1)

The paraoxonase enzyme produced in the human liver is a calcium dependent enzyme able to hydrolyze aryl esters, lactones, and OPs (Rajkovic et al., 2011). Structurally, it is also described as a six-bladed propeller structure that utilize a calcium ion within its active site similar to DFPase (Gold et al., 2000; Rajkovic et al., 2011; Mackness and Mackness, 2015). Although PON1 shares identity with DFPase in the active site and tertiary structure, it shows a different substrate preference and is unable to hydrolyze bulky OPs such as DFP but does show some activity for VX (Bajaj et al., 2013; Purg et al., 2017). In addition to its activity against organophosphates, paraoxonases have been extensively studied for their anti-inflammatory, anti-oxidative, anti-atherogenic, antimicrobial, anti-diabetic, and OP-detoxifying properties. Human paraoxonase-1 (PON1) can readily hydrolyze paraoxon, a commercial OP pesticide; however, its activity toward G- and V- type nerve agents is limited to the less toxic enantiomers of these agents. As a result, the enzyme has been subjected to substantial experimental and computational characterization to enhance its catalytic efficiency and enantioselectivity (Gupta et al., 2011; Goldsmith et al., 2012; Le et al., 2015).

As the only human-derived enzyme capable of degrading OP compounds, PON1 has the greatest potential as a therapeutic to counter OP poisoning. Unfortunately, PON1 suffers from a lack of stability unless bound to a high density lipoprotein (HDL) and is further limited (as this complex) by environmental conditions and by several common biomolecules that have been shown to interact with and limit the activity of the HDL-PON1 complex (Ferretti et al., 2001; Jaouad et al., 2003; Rozenberg and Aviram, 2006; Gaidukov et al., 2009). To improve upon the potential of PON1 as therapeutic, Aharoni et al. generated a mutant version of the PON1 that exhibited a 40-fold increase in OP-degrading activity compared to the parental enzyme (Aharoni et al., 2004). This recombinant PON1 was later combined with a HDL particle (referred to as BL-3050) and tested *in vivo* in mice by the same research team (Gaidukov et al., 2009). In these studies the recombinant PON1 showed a longer half-life in mouse models and provided a significant improvement in survival rates for treated mice (87.5%) vs. non-treated controls (37.5%).

### Organophosphate Hydrolase (OpdA)

One of the most efficient OP-degrading enzymes, organophosphate hydrolase (OpdA), was isolated form *Agrobacterium radiobacter* by Horne et al. in 2002 (Horne et al., 2002). The OpdA enzyme adopts an (α/β)_8_ barrel structure with a heterobinuclear Fe–Zn metal center and shows an enhancement in specific activity when supplemented with cobalt (Jackson et al., 2008). The enzyme can hydrolyze a wide variety of OP pesticides and has been shown to inactivate G-type nerve agents such as tabun (GA), sarin (GB), soman (GD), and ethylsarine (GE) with varying efficiencies (Dawson et al., 2008). Several mutants of OpdA with improved activity against these nerve agents have also been produced as described by Yang et al. (2003). In addition to *in vitro* studies, the *in vivo* therapeutic potential of OpdA has also been demonstrated. For instance, separate research groups showed that administration of OpdA in rats and monkeys improved survival after poisoning with highly toxic OP pesticides (Bird et al., 2008; Jackson et al., 2014). In addition to its therapeutic applications, OpdA is the only enzyme that is currently commercially employed as a bioremedier. The Australian company Orica Ltd, has marketed the OpdA-containing product Landguard™ OP-A for use in pesticide decontamination of water sources (Anderson et al., 2011; Scott et al., 2011). Studies starting in 2004 have shown that Landguard™ is able to significantly reduce OP levels in agricultural wastewater validating recombinant enzymes as a viable method of decontaminating contaminated sources at a significant scale.

### Phosphotriesterase (PTE)

Phosphotriesterase (PTE), also termed organophosphate hydrolase (OPH) is a zinc-dependent bacterial enzyme that belongs to the amidohydrolase superfamily and was first identified in soil bacteria that hydrolyzed the pesticide parathion (Dumas et al., 1989a,b). The tertiary structure of the protein is an (α/β)_8_-barrel or TIM-barrel, in which the binuclear active site is located at the C-terminus of the protein (Vanhooke et al., 1996; Benning et al., 2001). The most commonly utilized PTE enzyme was derived from *Brevundimonas diminuta* (previously *Pseudomonas diminuta*) although homologs have been identified in other species including *Sulfolobus solfataricus* (discussed below) and *Deinococcus radiodurans* which are typically referred to as phosphotriesterase-like lactonases (PLLs) (Dumas et al., 1989a; Merone et al., 2005; Hawwa et al., 2009). The microbial substrate of PTE and PLLs has not been identified, though it likely varies between species. It is believed and has been shown in some experimental studies that these enzymes likely arose from bacterial lactonases (Chow et al., 2009; Afriat-Jurnou et al., 2012; Elias and Tawfik, 2012). PTE shows high catalytic activity toward a number of pesticides but only moderate activity toward nerve agents such as sarin, cyclosarin, and VX. The environmental persistence of V-agents such as VX has led several research teams such as those of Frank Raushel of Texas A&M University and others to invest heavily in mutational studies of this enzyme to improve activity toward VX and other substrates (Briseno-Roa et al., 2011; Tsai et al., 2012; Bigley et al., 2013, 2015; Naqvi et al., 2014; Bigely et al., 2019). Recently, Khersonsky et al. developed a robust automated method which led to generation of PTE with a remarkable >1,000-fold improvement in nerve-agent hydrolysis (Khersonsky et al., 2018). Moving forward in that direction, Bigely et al. (2019), screened a 28,800 member six-site PTE mutant library against multiple V-agent analogs, and identified multiple variants with >1500-fold increase in *k*_cat_/*K*_M_ for the hydrolysis of VX (Bigely et al., 2019).

Of the OP-degrading enzymes currently studied, PTE has some of the fastest catalytic rates and has shown the greatest promise for engineering substrate activity as described above. Early studies with direct injection of PTE as a counter-measure for OP poisoning showed some success though the half-life of enzyme was relatively low (100 min) necessitating the development of an extracorporeal reactor system to purify the blood (Masson et al., 1998). A later review by Raushel summarized some of the progress with PTE as a therapeutic in mouse models including efforts to use PTE as a pretreatment to afford some level of protection (Ghanem and Raushel, 2005). With the mutational improvement of the enzyme's stability and substrate range it is not difficult to envision PTE as a viable medical countermeasure for OP poisoning.

### SsoPox

A hyperthermophilic PLL, SsoPox, was isolated from the Archaeon S*ulfolobus solfataricus* (Merone et al., 2005; Hiblot et al., 2012). SsoPox exhibits high activity toward acyl-homoserine lactones and oxo-lactones but a low phosphotriesterase activity. Similar to OP hydrolases of the amidohydrolase superfamily, the folded structure of SsoPox is an (α/β)_8_ barrel in which the active site resides at the C-terminal section of the structure (Hiblot et al., 2013). While SsoPox show reduced activity toward many pesticides compared to PTE and others, it is an extremely rugged enzyme exhibiting activity at temperatures up to 100°C (melting temperature is 104°C) and in the presence of several detergents and other denaturing agents (Hiblot et al., 2012). These properties make SsoPox a promising candidate for the development of field-deployable reagents for bioremediation. To further improve upon the enzyme, several groups have employed a structure-based design approach to increase the phosphotriesterase activity and improve the active site recognition for an increased range of OP substrates (Merone et al., 2010; Jacquet et al., 2017). Recently, Vitola et al. developed a biocatalytic membrane reactor (BMR) by covalently immobilizing a triple mutant of the SsoPox on polymeric membranes, leading not only to high paraoxon degradation but also long-term stability of the free enzyme (Vitola et al., 2019).

### Organophosphate Acid Anhydrolase (OPAA)

The organophosphate acid anhydrolase enzymes are bacterial prolidases isolated from several species of *Alteromonas* bacteria (deFrank and Cheng, 1991). The *Alteromonas* OPAAs are dipeptidases that typically cleave dipeptide bonds in which the C-terminal residue is a proline (Cheng et al., 1999). Although not involved in the metabolism of OPs, OPAAs also show activity against a wide range of these compounds cleaving P-F, P-O, P-CN, and P-S bonds (Cheng et al., 1999). Prolidases are structurally distinct from other bacterial hydrolases such as PTE and OpdA and therefore show different OP substrate specificities and activities (Xiao et al., 2017). As with many of the other enzymes discussed here, OPAA has undergone mutagenic studies to enhance its catalytic activity and substrate specificity. Bae *et al*. showed that a series of mutations in OPAA led to significant increase in activity toward sarin and soman (Bae et al., 2018). Additionally, in these studies, the authors were able to isolate a double mutant version of OPAA that was able to show enantioselectivity for one enantiomer of sarin. The *Alteromonas* OPAA, similar to other OP enzymes, have greatest activity at biologically-relevant temperature (25–37°C) which can limit their utility in field-based applications. As has been observed with other proteins, prolidases isolated from extremophiles such as members of the *Pyrococcus* genus often show improved thermostability as shown by Theriot et al. (2011). Studying both the wild-type and mutant versions of the *Pyrococcus horikoshii* enzyme, the authors showed that these new OPAA enzymes had improved shelf-life and thermal stability compared to *Alteromonas* OPAAs making them ideal candidates for field distribution.

### Other Enzymes

In addition to the above described enzymes, several proteins have been shown to possess a moonlighting OP hydrolyzing activity. For instance, senescence marker protein (SMP30) isolated from mouse liver cytosol, is capable of hydrolyzing DFP and other OPs such as sarin, soman, and tabun (Scott and Bahnson, 2011; Dutta et al., 2019). Although SMP30 can hydrolyze similar substrates like DFPase, it is not a calcium binding protein, and shows activity in the presence of Mg^2+^ and Mn^2+^ (Dutta et al., 2019). Bacterial lactonases have also shown potential as OP-degrading enzymes due to their high degree of substrate promiscuity (Draganov, 2010). Like SsoPox, other PLLs have been described in the literature as showing some potential as tools for OP bioremediation. In separate publications, Zhang et al. describe a PLL from *Geobacillus kaustophilus* referred to as GkaP (Zhang et al., 2012, 2015). This enzyme typically cleaves the 6-membered ring structures of lactones but does show some activity toward *ethyl*-paraoxon. Other enzymes capable of degrading the substrate methyl-parathion have also been described in the literature (Rani and Lalithakumari, 1994; Zhongli et al., 2001; Yang et al., 2008). These enzymes typically utilize a binuclear metal active site but show little structural similarity to phosphotriesterases (Dong et al., 2005).

Many of the enzymes presented here have a long history in the development of therapeutics, sensors, and agents for environmental and personal decontamination. As the tools of genomics and systems, synthetic, and molecular biology continue to evolve it is anticipated that more enzymes capable of degrading OP compounds will be identified in nature and evolved in the laboratory to improve their applicability. In the subsequent sections we discuss the efforts of researchers to both improve and implement these enzymes for field use. As a discussion encompassing all of the aforementioned enzymes would prove prohibitively long, we focus on efforts targeting the PTE or OPH enzymes only.

## Relevance and Potential for Deployment

OP compounds fill two distinct and highly divergent applications in human society. At the most benign level, the compounds are used to benefit agricultural production and to control the spread of arthropod disease. At the opposing end of this spectrum, OP compounds are some of the most dangerous weapons produced by man. Though the potential for human harm is dramatically different, even widely used pesticides can be incredibly dangerous following prolonged or high concentrations of exposure. Therefore, the need to develop reliable methods to both detect and eliminate these compounds is important and relevant to both civilian and military populations. While successes have been achieved with engineering bacterial systems for decontamination and detection through mechanisms such as cell-surface localization of enzyme, the release of engineered organisms is not currently accepted by nations around the world. Cell-free synthetic biology, which can rely on cell extracts or purified recombinant enzymes, offers an avenue for utilizing the elegance of biological systems for detection and bioremediation in a non-replicating system. As will be described in the subsequent sections, enzyme-based cell-free systems have seen success as sensors, tools of bioremediation, and as potential therapeutics.

### Sensors

**Figure 5:**
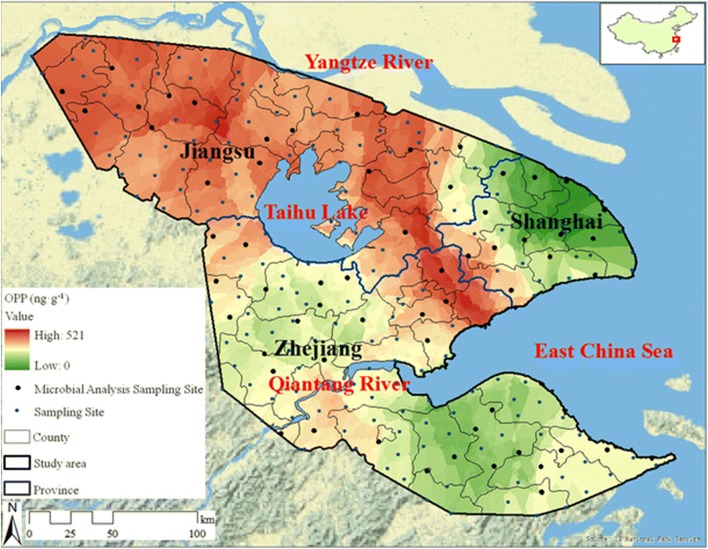
Spatial distribution of organophosphate concentrations across the Yangtze River Delta region. Researchers tested soil samples (241 sites covering 45,800 km^2^) from the Yangtze River Delta to assess the spatial distribution of OP pesticides (and others) due to agricultural use. The representative map shown here highlights 60 sites with a distribution of contamination levels. Reproduced with permission (Pan et al., 2018), Copyright Springer 2018.

While the use of pesticides is vital to commercial agriculture, the long half-lives and soil and water retention time of many of these organophosphate compounds necessitates environmental monitoring (Uchimiya et al., 2012; Hossain et al., 2014; Fosu-Mensah et al., 2016; Pan et al., 2018). Contamination of water sources by agricultural run-off leads to elevated concentrations of these compounds in large-scale waterways such as rivers and deltas as well as soil (Pedersen et al., 2006; Babu et al., 2011). Often, OP compounds are photodegradable or easily hydrolyzed in aerobic soils, however, as shown in a recent publication by Pan et al. and the map of the Yangtze River Delta region shown in Figure 5, elevated concentrations of OP compounds can easily be measured in environmental samples (Pan et al., 2018). While these effects are largely felt at the local level, the globalized agricultural economy enables contaminated food products to be distributed world-wide.

The need for OP sensors touches both civilian and military walks of life. PTE-based sensors have not transitioned beyond the laboratory, however, several research groups have shown that surface-immobilized PTE is viable in sensor development (Schoning et al., 2003; Simonian et al., 2004; Andrianova et al., 2016; Hondred et al., 2017, 2018). Typically, these sensors are electrochemical in nature monitoring the formation of hydrolysis products. Schöning et al. explored a number of parameters such as immobilization strategies and reaction conditions while developing a sensor which measures small changes in pH resulting from the hydrolysis of OP compounds by PTE (Schoning et al., 2003). As another example, Andrianova et al. describe an ion-selective field-effect transistor (ISFET) that utilized immobilized PTE to detect paraoxon and methyl-paraoxon (Andrianova et al., 2016). In these studies the researchers highlighted the utility of their ISFET system in prolonged and continuous monitoring of water samples without loss of function which is relevant to the development of distributable water system sensors.

**Figure 6:**
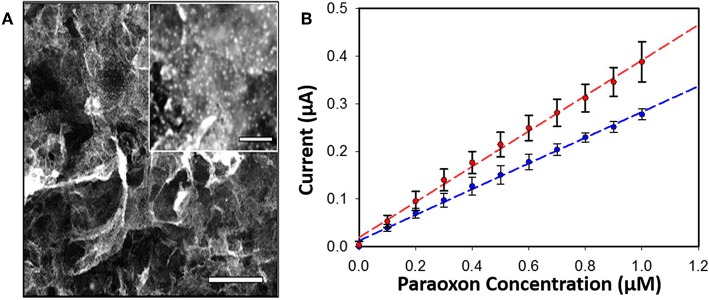
PTE-based sensor for OP compounds. An amperometric sensor was developed that utilized PTE-coated platinum NPs laser-annealed to a graphene-coated electrode for detection of the OP pesticide paraoxon (**A**, inset show platinum particles, scale bar 200 nm). Amperometric current was monitored with and without the graphene coating **(B)** showing measurable improvement between the two systems. Reproduced with permission from Hondred et al. (2018), Copyright the American Chemical Society 2018.

Enzyme concentration and activity in sensors is vitally important to achieving the high levels of sensitivity desired in OP detection. Often this can be accomplished through the integration of nanoparticles into the sensor platform as a scaffold for enzyme assembly. In two separate studies Hondred et al. developed a printed, graphene-based sensor to detect paraoxon and other pesticides highlighting the use of both enzymes and electrochemical detection (Hondred et al., 2017, 2018). The researchers used a variety of manufacturing techniques including the laser annealing of platinum nanoparticles (NPs) to increase enzyme loading and the overall sensitivity of the sensor as shown in Figure 6. In these studies they were able to show a detection limit of 3 nM for the paraoxon substrate. The benefits of combining nanostructures and enzymes in OP sensors has been realized and exploited by several other research groups whose efforts are summarized in a recent review by Sheng et al. (Xiong et al., 2018).

### Improving the Utility of PTE

As discussed above for each of the OP hydrolases, protein engineering methods have led to significant improvement in the activity of the enzyme and substrate/enantio- specificity. Beyond direct modification of the amino acid sequence, immobilization of enzymes to solid supports further improves their applicability as there are often inherent benefits of improved stability and enhanced catalytic activity associated with immobilization (Mateo et al., 2007; Mohamad et al., 2015; Proschel et al., 2015; Hoarau et al., 2017). As an enzyme with significant potential as both a bioremedier and therapeutic, PTE has been attached to a range of different materials to improve its activity, long-term stability, and; therefore, viability as a deployable reagent. To illustrate the potential for increased functionality of PTE when immobilized to a solid surface, Raynes et al. cross-linked the enzyme to amyloid fibrils generated from insulin and crystallin and observed a marked improvement in thermal stability (Raynes et al., 2011). Similarly, covalently conjugating PTE to negatively charged gold nanoparticles not only decreased Km value and increased Vmax and Kcat values, but also achieved stability within a wider range of pH (2–12) and temperature (25–90°C) (Karami et al., 2016). This phenomenon of improved enzymatic and biophysical properties is not unique to PTE as highlighted in numerous review articles and recent publications (Chen M. et al., 2017; Kreuzer et al., 2017; Arsalan and Younus, 2018; Wang et al., 2018).

**Figure 7:**
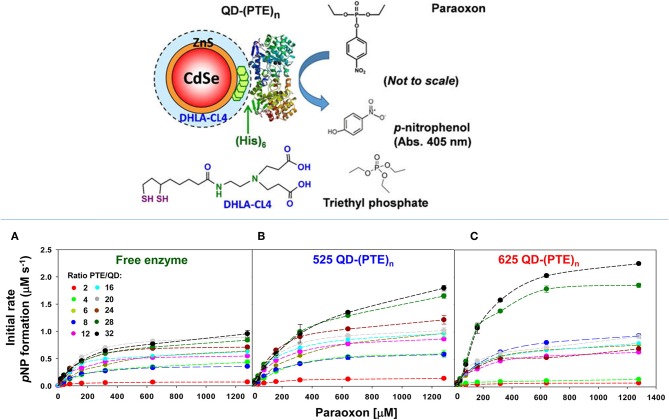
Enhancing PTE activity using PTE-QD conjugates. Researchers have seen an increase in the catalytic activity of PTE when immobilized to the surface of QD NPs. In studies described by Breger et al., PTE was attached to CdSe NPs using an oriented assembly of the enzyme via a C-terminal His_6_ amino acid sequence (schematic in the upper panel). Here the hydrolysis of paraoxon by PTE was monitored at 405 nm to assess enzyme activity for both free PTE and PTE immobilized to two QD NPs. Significant changes in the rate of initial velocity for PTE were observed when the enzyme was immobilized to 525 nm **(B)** and 625 nm **(C)** QDs. The rate of catalysis for free enzyme **(A)** was compared to each PTE-QD structure at varying ratios of nanoparticle to enzyme. In each instance the rate of reaction was markedly improved compared to the free enzyme control. Reproduced with permission (Breger et al., 2015a), Copyright American Chemical Society 2015.

**Figure 8:**
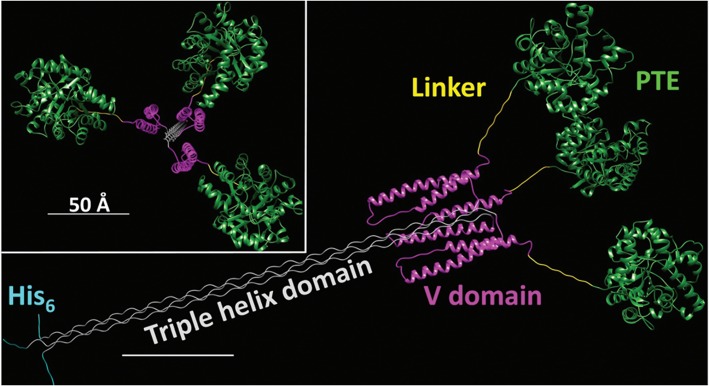
Schematic of an Engineered PTE Triple Helix Structure**—**A bacterial collagen-like domain was appended to the end of PTE to force the assembly of a trimer structure with a rigid tail section that could be assembled to QDs and other NPs for the purpose of studying kinetics of this non-natural structure. Reproduced from Breger et al. (2015b) with permission from the Royal Society of Chemistry.

The enhancement of PTE activity and other enzymes at nanoparticle interfaces has been a focus of research efforts in the Medintz laboratories for several years. Focusing on semiconductor quantum dots (QDs) as the model scaffold, the team developed strategies for QD capping and PTE immobilization that led to the formation of highly active enzyme structures (Susumu et al., 2014). A subsequent study described in Breger et al., showed that the rate and efficiency of enzyme catalysis was directly affected by the size of the QD NP as shown in Figure 7 (Breger et al., 2015a). While PTE is a highly active enzyme functioning at nearly the rate of diffusion, the team observed an ~4-fold increase in the initial rate of the reaction and an increase in efficiency ~2-fold greater than the free enzyme. Further experiments showed that this enhancement was not the result of a reduction in activation energy rather a change in the rate of dissociation of product from the enzyme active site as similarly described for the free enzyme by Caldwell et al. (1991). Expanding upon these successes, the team assembled a trimeric PTE structure by attaching a collagen-like domain to the C-terminus of this enzyme (Breger et al., 2015b). The goal here was to create a material that could be spun into a fiber for subsequent integration into textiles such as clothing. Efforts focused on both increasing the potential packing density of the enzyme as well as examining the effect on enzyme activity as the spatial positioning of the enzyme increased in relation to the QD surface as seen in the schematic of Figure 8. Though the overall activity of the PTE-trimer was reduced compared to monomeric PTE, the QD-assembled, trimeric structures did show enhanced activity compared to free trimer when assembled to the NPs. The authors postulated that the observed enhancement could be the result of substrate localization due to the expanded solvation shell, the increased avidity due to the trimeric structure, or the controlled orientation of the enzyme itself.

**Figure 9:**
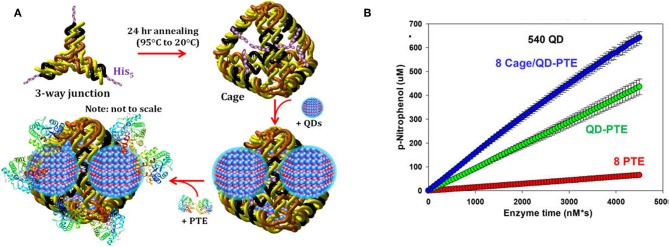
Enhancement of PTE activity through scaffolded DNA assemblies. Pyramidal DNA cages were assembled using three-way DNA junctions that presented terminal hexahistidine sequences allowing for the positional assembly of QDs and recombinant PTE **(A)**. The initial velocity of each construct was tested in parallel **(B)** with “8 PTE” correlating to 8 free enzymes, “QD-PTE” correlating to 8 enzymes on each 540 nm QD, and “8 cage/QD-PTE” defining the assembled structure of **(A)**. Reproduced with permission (Samanta et al., 2018), Copyright the American Chemical Society 2018.

As has been shown with QDS and other abiotic scaffolds, immobilization of enzymes to solid supports is beneficial in sensor development and, in many cases, efforts to improve the kinetic and biophysical properties of the enzyme. To ensure optimum benefit from immobilization it is imperative to ensure enzyme function by carefully selecting immobilization strategies that (1) ensure the enzyme structure is not perturbed and (2) the active site of the enzyme is not occluded in any way. DNA scaffolds and DNA nanostructures can be computationally designed based on Watson-Crick base pairing to ensure consistent and reproducible display of enzymes (Proschel et al., 2015; Quin et al., 2017; Qiu et al., 2018; Sun et al., 2019). Using QDs as their solid support and PTE as their model enzyme, Breger et al. designed a strategy to conjugate the two components using a DNA linker comprised of three modified single-stranded DNA (ssDNA) oligomers (Breger et al., 2017). The goal here was to establish novel methods of QD-functionalization that potentially reduced biomolecule fouling of the QD surface while simultaneously validating a method that could be used for subsequent spatial positioning studies. Conveniently, QDs provide a simple method of biomolecular immobilization via metal affinity interaction between the shell of the QD and hexahistidine (His_6_) residues at the enzyme's termini which are included for purification over nickel chelate media. The PTE was conjugated to a second ssDNA molecule and then tethered to the QD surface using a third ssDNA molecule that was complementary to the other two sequences. In these studies, the authors observed some variability in the rate enhancement between conditions but were able to conclusively show that overall enzyme activity was improved above that of free enzyme using this method of NP assembly. In a subsequent study described by Samanta et al., the research team altered their strategy using a pyramidal DNA structure as a scaffold to arrange PTE-coated QDs on the surface of the DNA cage in hopes of establishing a new hybrid scaffolding system to enhance enzyme activity (Samanta et al., 2018). Here the authors designed ssDNA oligos, each terminating with a trimeric histidine repeat that would assemble into 3-way junctions. These subunits were further combined to form a pyramidal DNA cage. The free histidine repeats were then used to assemble QDs to the cage surface through metal affinity between the QD and the terminal histidine amino acids. Subsequent attachment of PTE was accomplished as previously described using the Hi_6_ tag at the C-terminus of the enzyme see Figure 9. As in previous efforts by the Medintz research team, variability in enzyme enhancement was observed based on the size of the QD and on the ratio of enzyme and the DNA cages. The optimized conditions in these studies showed a nearly 12.5-fold enhancement in the rates of catalysis for the DNA cage, QD-assembled enzyme compared to both free enzyme controls and PTE assembled to QD alone.

### Enabling PTE Deployment

Enzymes are notoriously limited by issues of stability. Once removed from the ideal biological conditions of the cell, these intricately folded structures often unravel or collapse into non-functional amino acid chains or globules. While loss of function is expected with all biomolecules, researchers and commercial entities alike struggle to develop methods to limit loss of enzyme activity through storage formulation, direct protein engineering, and other approaches. The efficiency of remediation, rapid rates of catalysis, and environmental and biological compatibility of OP hydrolases such as of PTE make these ideal candidates for the development of commercial products. As mentioned above attaching enzymes to solid supports has been shown to improve enzyme stability and longevity. These strategies, however, are not always amenable to the development of therapeutic agents nor the wide-spread distribution of the materials due to cost of production and the potential for secondary immune response or environmental contamination. In contrast, the encapsulation of enzymes and other biomolecules has a long history in the development of therapeutics and, as will be discussed below, a future in the development of enzyme systems for bioremediation.

While the human PON1 enzyme shows catalytic activity against both V- and G-type agents, the rates of catalysis are not high enough for this enzyme to be an ideal therapeutic or prophylactic (Rajkovic et al., 2011; Le et al., 2015). In contrast, PTE catalysis of many OP compounds occurs at the rate of diffusion (Ghanem and Raushel, 2005). In a foundational publication, Pei *et al*. developed a method of PTE encapsulation targeting implementation of the enzyme as a medical counter measure (Pei et al., 1994). Here the authors permeablized murine erythrocytes which were subsequently filled with recombinant PTE using a hypotonic solution then resealed. This method yielded between 30 and 77% encapsulation depending upon the methods employed. While the PTE-filled erythrocytes were not tested *in vivo* the authors did show that hydrolysis of paraoxon could be achieved using this system and that enzyme encapsulation did not impair enzyme function. Budai et al. shifted away from natural carriers and characterized enzyme activity in synthetic stealth liposomes showing efficient hydrolysis of paraoxon in *in vitro* studies (Budai et al., 2009). In these foundational studies, the research team examined a range of lipid monomers and assessed overall stability of liposome-enzyme construct over 4 days in guinea pig plasma. The authors showed that the encapsulated enzyme was able to significantly out-perform the free enzyme in both plasma and controls in buffered solutions. Liposomal encapsulation has also been used for the colocalization of enzyme and other therapeutics such as atropine as described for OPAA by Petrikovics (Petrikovics et al., 2000). These studies suggest that encapsulation of enzyme could be used as pathways for *in vivo* delivery of detoxifying therapeutics, or as discussed below, vehicles for field deployment.

**Figure 10:**
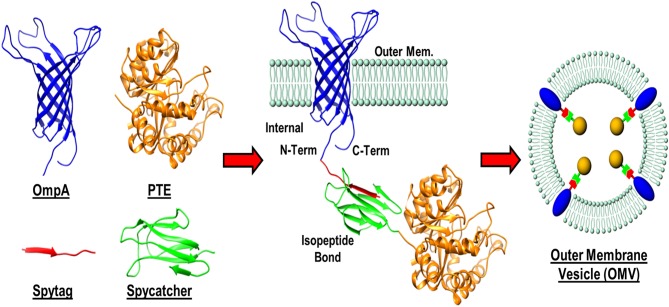
Protein-protein ligation strategy for outer membrane vesicle (OMV) loading. A recombinant membrane protein (OmpA) presenting a SpyTag label was expressed in *E. coli* as an outer membrane anchor for a phosphotriesterase (PTE)—SpyCatcher fusion protein. Interaction between the SpyTag/SpyCatcher partners allowed for the PTE enzyme to be pulled in to nascent OMVs during formation.

**Figure 11:**
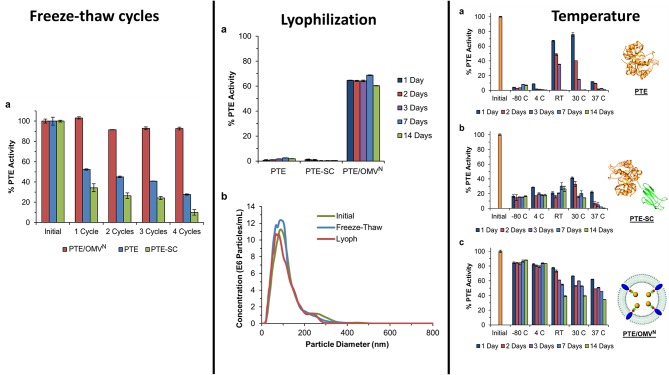
Encapsulation of PTE within bacterial OMVs**—**The directed packaging of PTE within bacterial OMVs conveys improved stability to the enzymes allowing it to maintain activity over several common storage conditions such as freezing at −80°C and lyophilization and rehydration. Unlike some synthetic liposome formulations, bacterial OMVs maintain their morphology following lyophilization and rehydration (middle panel, b). Encapsulation within the vesicles also allowed the enzyme to be stored for up to 14 days over a range of conditions while maintain a high level of enzyme activity compared to controls. This figure is a combination of data sets presented in Alves et al. (2015, 2016). Both figure reproduced with permission.

In addition to enzyme stability, the cost of production can often be limiting to the realization of enzyme-based tools for bioremediation. In efforts to circumvent both of these complications simultaneously, researchers at the U.S. Naval Research Laboratory explored the direct production and encapsulation of PTE in proteoliposomes produced by *Escherichia coli*. Throughout their life cycle, bacteria release fragments of their outermost membrane containing proteins, nucleic acids, and other biomolecules which have been implicated in a range of functions from pathogenesis to community communication (Deatherage et al., 2009; Schwechheimer et al., 2013). As mimics of the parental bacterium's membrane, outer membrane vesicles (OMVs) are comprised of a protein decorated lipid bilayer that affords protection to cargo proteins from environmental proteases and nucleases. To exploit these benefits, Alves et al. utilized a protein-protein ligation system to anchor PTE to the inner wall of the *E. coli* outer membrane enabling protein loading into nascent OMVs, see Figure 10 (Alves et al., 2015). Purified directly from the bacterial culture media, these particles showed high catalytic activity toward paraoxon. In subsequent studies, the research group showed that these biological NPs conveyed significant improvements to the stability of the encapsulated PTE allowing it to undergo iterative cycles of freezing and thawing and lyophilization as shown in Figure 11 (Alves et al., 2016). Additionally, enzyme activity was preserved under elevated temperatures, in high salt solutions that mimic seawater, and over a range of pH conditions (Alves et al., 2016, 2018). These PTE-filled OMVs were also tested in paraoxon-spiked environmental water samples, on surfaces such as painted metal coupons produced to mimic military vehicles, and on glass surfaces to demonstrate their utility in bioremediation (Alves et al., 2018). Finally, OMVs for PTE production and utilization also allowed for a simple and cost-effective method of large-scale enzyme production and dissemination. Alves et al. showed that their PTE-filled OMVs could also be easily purified from bulk growth media using an engineered His_6_ affinity tag expressed on the surface of the vesicle (Alves et al., 2017). As bacteria such as the *E. coli* used here can be grown in fermenters at industrial scale, this one step purification protocol significantly reduces the burden associated with manufacture and downstream processing often associated with commercial enzyme production.

Taken as a whole and shown here with PTE, OMVs offer a highly adaptable vehicle for the design, development, and implementation of green biological tools for bioremediation. The ease of production using fermentation currently employed by large- and small-scale breweries shows the feasibility of this form of biomanufacturing. Additionally, the improvements to storage and enzyme stability has been observed with other OP-degrading enzymes such as DFPase and others (unpublished data from the Walper laboratory). Finally, OMVs offer a highly versatile platform to engineer and develop new bioremediation tools. The Walper laboratory has focused on *E. coli* as their proof-of-concept platform but other environmental species could similarly be engineered to develop bioremediation tools. As non-replicating biological particles, upstream modification of membrane channels, surface features, or others to improve functionality would likely not fall under the same regulations for environmental release currently encountered for microbial species. In conclusion, the composition of bacterial membrane vesicles enables researchers to devise numerous strategies for both their functionalization and purification which will undoubtedly lead to the development of new technologies exploiting these materials as sensors and tools for environmental decontamination (Chen Q. et al., 2017; Jan, 2017; Su et al., 2017).

## The Future of Enzymatic Systems for Bioremediation and Decontamination

The purpose of this review to was to illuminate the reader to both the need for, and successes with enzymes as tools for the bioremediation and decontamination of OP compounds. Whether used as pesticides or agents of war, these compounds have demonstrated potential to cause both acute and long-term harm to individuals and ecosystems alike. Unfortunately, with their wide-scale use in both developed and developing countries it is impossible to foresee a future in which these compounds are not released upon the world at a scale of millions of tons per annum. While chemical methods, and physical methods to some degree, can be employed for decontamination, neither of these pathways is sustainable. Therefore, it is imperative to establish green and sustainable bioremediation tools.

As we have shown here, scientists continue to make significant advances in developing enzymes as tools for environmental and personnel decontamination. In this review we very briefly describe six of the more prominent enzymes capable of degrading organophosphate compounds, however, with increasing genomic sequencing and cataloging of environmental microbes researchers will undoubtedly tease out new enzymes and novel pathways for the bioremediation of OPs and other environmental contaminants. As shown here, using the tools of molecular biology and protein design researchers have been able to significantly modify these enzymes vastly increasing the substrate binding ability and catalytic activity. These foundational studies will continue to evolve to improve both current and future OP hydrolases.

The large-scale use of enzymes as commercial products, therapeutics, and others has historically been impeded by the costs and difficulties associated with manufacture and stabilization. While these factors have historically proved as arguing points, companies such as Novozyme and others continue to demonstrate that the commercialization of enzymes in attainable and profitable. Additionally, the rapid proliferation of synthetic biology-based research efforts have led to the formation of companies such as Gingko Bioworks that focus largely on improving biomanufacturing capabilities through protein selection, gene optimization, and large-scale fermentation models. With significant financial backing in these companies and others, it is undeniable that there is a growing recognition in the potential of biological systems.

Finally, we have shown here with examples such as QD scaffolds and OMV encapsulation that downstream processing of enzyme systems offer potential mechanisms of significantly improving the applicability of enzymes as field deployable materials. In the proof-of-concept studies shown here we see significant improvements in both catalysis and stability using assembly and encapsulation platforms. These systems also offer the potential for multi-enzyme assemblies to move beyond the simple hydrolysis of substrate “A” to product “B,” but rather from “A” to “D” through multi-enzyme pathways that demonstrate increased catalytic activity due to substrate channeling or proximity. These multi-enzyme pathways will be necessary to completely eliminate organophosphate compounds which are often nearly as toxic as the first hydrolysis product (Council, 1996).

In conclusion, while significant gains have been made in developing enzymes as candidate biomaterials the road ahead is still a long one. Further demonstrations in relevant environments and conditions are required to validate the potential of the materials for both personal and large-scale decontamination alike. This will require active collaborations between academy, government, and industry; each of which possesses a unique set of skills that can contribute to the realization of enzymes as tools for bioremediation and in decontamination. Unfortunately, even if all of these pieces are combined and the potential of enzymes realized, it is changes in societal and institutional perceptions of these dangerous compounds and their long-term impacts that are most needed and likely the furthest off.

## Author Contributions

All authors listed have made a substantial, direct and intellectual contribution to the work, and approved it for publication.

### Conflict of Interest

The authors declare that the research was conducted in the absence of any commercial or financial relationships that could be construed as a potential conflict of interest.
