# Supplementary material for: Enzymatic Bioremediation of Organophosphate Compounds—Progress and Remaining Challenges
Source: Front Bioeng Biotechnol. 2019 Nov 8;7:289. doi: 10.3389/fbioe.2019.00289 (PMC6856225; doi:10.3389/fbioe.2019.00289)
Supplement: Supplementary file 1 [file Table_1.docx]

**Supplemental material**

**Enzymatic Bioremediation of Organophosphate Compounds – Progress and Remaining Challenges**

Meghna Thakur^1^, Igor L. Medintz^2^, and Scott A. Walper^2*^

^1^ College of Science, George Mason University, Fairfax, VA 22030, USA

^2^ Center for Bio/Molecular Sciences, U.S. Naval Research Laboratory, Washington, D.C. 20375

^*^ author to whom correspondence should be addressed [scott.walper@nrl.navy.mil](mailto:scott.walper@nrl.navy.mil)

**Running Title –** Enzymes for Organophosphate Bioremediation

**Supplemental Table1 – Organophosphate Insecticides Recognized by the US Environmental Protection Agency**

| ***Highly toxic compounds*** |  |  |
| --- | --- | --- |
| azinphos-methyl | Guthion, Gusathion |  |
| bomyl | Swat | No longer registered in US |
| carbophenothion | Trithion |  |
| Chlorfenvinphos | Apachlor, Birlane |  |
| Chlormephos | Dotan | No longer registered in US |
| Chlorthiophos | Celathion | No longer registered in US |
| Coumaphos | Co-Ral, Asuntol |  |
| Cyanofenphos | Surecide | No longer registered in US |
| Demeton | Syntox | Systemic toxin taken up by plants and retained in leaves and fruit |

| Dialifor | Torak |  |
| --- | --- | --- |
| Dicrotophos | Bidrin |  |
| Dimefos | Hanane, Pestox XIV |  |
| Dioxathion | Delnav |  |
| Disulfoton | Disyston | Systemic toxin taken up by plants and retained in leaves and fruit |
| Endothion |  | No longer registered in US |
| EPN |  |  |
| Ethyl parathion | E605, Parathion, Thiophos |  |
| Famphur | Famfos, Bo-Ana, Bash | No longer registered in US |
| Fenamiphos | Nemacur |  |
| Fenophosphon | Trichloronate, Agritox | No longer registered in US |
| Fensulothion | Dasanit |  |
| Fonofos | Dyfonate, N-2790 |  |
| Fosthietan | Nem-A-Tak |  |
| Isofenphos | Amaze, Oftanol |  |
| Mephosfolan | Cytrolane | No longer registered in US  Systemic toxin taken up by plants and retained in leaves and fruit |
| Methamidophos | Monitor |  |
| Methidathion | Supracide, Ultracide |  |
| Methyl parathion | E601, Penncap-M |  |
| Mevinphos | Phosdrin, Duraphos |  |
| Mipafox | Isopestox, Pestox XV | No longer registered in US |
| Monocrotophos | Azodrin |  |
| Phorate | Thimet, Rampart, AASTAR |  |
| Phosfolan | Cyolane, Cylan | No longer registered in US  Systemic toxin taken up by plants and retained in leaves and fruit |
| Phosphamidon | Dimecron |  |
| Prothoate | Fac | No longer registered in US  Systemic toxin taken up by plants and retained in leaves and fruit |
| Schradan | OMPA | No longer registered in US |
| Sulfotep | Thiotepp, Bladafum |  |
| Terbufos | Counter, Contraven |  |
| Tetraethyl pyrophosphate |  | No longer registered in US |
| ***Moderately toxic compounds*** |  |  |
| Acephate | Orthene |  |
| Bensulide | Betasan, Prefar |  |
| Bromophos-ethyl | Nexagan | No longer registered in US |
| Bromophos | Nexion | No longer registered in US |
| Chlorphoxim | Baythion-C | No longer registered in US |
| Chlorpyrifos | Dursban, Lorsban, Brodan |  |
| Crotoxyphos | Ciodrin, Cypona |  |
| Crufomate | Ruelene | No longer registered in US |
| Cyanophos | Cyanox | No longer registered in US |
| Cythuiate | Proban, Cyflee | No longer registered in US |
| DEF | De-Green, E-Z-Off D |  |
| Demeton-S-methyl | Duratox, Metasystox-R | Systemic toxin taken up by plants and retained in leaves and fruit |
| Diazinon | Spectracide |  |
| Dichlofenthion | VC-13 Nemacide |  |
| Dichlorvos | DDVP, Vapona |  |
| Edifenphos |  | No longer registered in US |
| EPBP | S-Seven | No longer registered in US |
| Ethion | Ethanox |  |
| Ethoprop | Mocap |  |
| Etrimfos | Ekamet | No longer registered in US |
| Fenitrothion | Accothion, Agrothion, Sumithion |  |
| Fenthion | Mecaptophos, Entex, Baytex, Tiguvon |  |
| Formothion | Anthio | No longer registered in US |
| Heptenophos | Hostaquick | No longer registered in US |
| IBP | Kitazin |  |
| Iodofenphos | Nuvanol-N | No longer registered in US |
| Isoxathion | E-48, Karphos | No longer registered in US |
| Leptophos | Phosvel | No longer registered in US |
| Malathion | Cythion |  |
| Merphos | Folex, Easy Off-D |  |
| Methyl trithion, dimethoate | Cygon, DeFend | No longer registered in US |
| Naled | Dibrom |  |
| Oxydemeton-methyl | Metasystox-R | Systemic toxin taken up by plants and retained in leaves and fruit |
| Phencapton | G 28029 | No longer registered in US |
| Phenthoate | Dimephthoate, Phenthoate | No longer registered in US |
| Phosalone | Zolone |  |
| Phosmet | Imidan, Prolate |  |
| Phoxim | Baythion | No longer registered in US |
| Pirimiphos-ethyl | Primicid | No longer registered in US |
| Pirimiphos-methyl | Actellic |  |
| Profenofos | Curacron |  |
| Propetamphos | Safrotin |  |
| Propyl thiopyrophsophate | Aspon | No longer registered in US |
| Pyrazophos | Afugan, Curamil | No longer registered in US |
| Pyridaphenthion | Ofunack | No longer registered in US |
| Quinalphos | Bayrusil | No longer registered in US |
| Runnel | Fenchlorphos, Korlan |  |
| Sulprofos | Bolstar, Helothion | No longer registered in US |
| Temephos | Abate, Abathion |  |
| Tetrachlorvinphos | Gardona, Apex, Stirofos |  |
| Thiometon | Ekatin | No longer registered in US |
| Triazophos | Hostathion | No longer registered in US |
| Trichlorfon | Dylox, Dipterex, Proxol, Neguvon |  |

* compounds listed in order of descending toxicity based on oral administration in rats.
